# Does Transition to Retirement Promote Grandchild Care? Evidence From Europe

**DOI:** 10.3389/fpsyg.2021.738117

**Published:** 2021-09-20

**Authors:** Antti O. Tanskanen, Mirkka Danielsbacka, Hans Hämäläinen, Aïda Solé-Auró

**Affiliations:** ^1^Department of Social Research, University of Turku, Turku, Finland; ^2^Population Research Institute, Helsinki, Finland; ^3^Department of Political and Social Science, Pompeu Fabra University, Barcelona, Spain

**Keywords:** childcare, Europe, grandparental investment, retirement, SHARE

## Abstract

Evolutionary theory posits that grandparents can increase their inclusive fitness by investing in their grandchildren. This study explored whether the transition to retirement affected the amount of grandchild care that European grandparents provided to their descendants. Data from five waves of the longitudinal Survey of Health, Aging, and Retirement in Europe collected between 2004 and 2015 from 15 countries were used. We executed within-person (or fixed-effect) regression models, which considered individual variations and person-specific changes over time. It was detected that transition to retirement was associated with increased grandchild care among both grandmothers and grandfathers. However, the effect of retirement was stronger for grandfathers than for grandmothers. Moreover, transition to retirement was associated with increased grandchild care among both maternal and paternal grandparents, but there was no significant difference between lineages in the magnitude of the effect of transition to retirement on grandchild care. In public debate retirees are often considered a burden to society but the present study indicated that when grandparents retire, their investment in grandchildren increased. The findings are discussed with reference to key evolutionary theories that consider older adults' tendency to invest time and resources in their grandchildren.

## Introduction

Grandparents share ~25% of the same genes with their grandchildren, meaning that older adults can increase their inclusive fitness by investing nurturance, protection, material support, and other resources in their grand-offspring (Hamilton, 1964). Grandparental investment is an extended version of parental investment (Trivers, 1972) and can be defined as any support that grandparents channel toward their grandchildren either directly, or indirectly via the grandchildren's parents (Coall and Hertwig, 2010). Grandparental investment is often measured as grandchild care, which indicates investment in time, care, and resources channeled toward descendants (Tanskanen and Danielsbacka, 2019). In contemporary societies, grandparents provide a significant amount of grandchild care; for instance, 56% of European grandparents look after grandchildren at least occasionally (Hank and Buber, 2009).

Grandparent types (i.e., maternal grandmothers, maternal grandfathers, paternal grandmothers, and paternal grandfathers) may not provide an equal amount of childcare to their descendants. Evolutionary scholars have predicted that biased grandparental investment is related to paternity uncertainty (Smith, 1991; Euler and Weitzel, 1996). Maternal grandmothers are the only ones who can be sure that their grandchildren are genetically related to them, while maternal grandfathers (via themselves and their daughters) and paternal grandmothers (via their sons and sons' children) have one link of paternity uncertainty, and paternal grandfathers have two uncertain links (via themselves and their sons and via their sons and sons' children). Moreover, because of sex-specific reproductive strategies (i.e., women carry babies for nine months and then breastfeed them), having children is typically more costly for women than for men (Trivers, 1972), meaning that maternal investment tends to be more obligatory, whereas paternal investment can be more facultative. Because of paternity uncertainty and sex-specific reproductive strategies, individuals are also predicted to be more willing to invest in maternal rather than paternal kin; hence, there is a matrilateral bias in kin investment (Daly and Perry, 2017; Perry and Daly, 2017). In line with the evolutionary predictions, several studies from contemporary Western societies have provided support for the sex- and lineage-based differences in grandparental investment showing that grandmothers invest more in grandchildren than grandfathers and maternal grandparents more than paternal grandparents (e.g., Smith, 1991; Euler and Weitzel, 1996; Laham et al., 2005; Pollet et al., 2006, 2007; Bishop et al., 2009; Danielsbacka et al., 2011).

An important factor shaping grandparents' opportunities to invest in grandchildren in contemporary societies is their employment status, that is, whether grandparents are retired or not. After retirement, grandparents have significantly more time to invest in grandchild care than when they were still working (Lakomý and Kreidl, 2015; Feng and Zhang, 2018). Although older adults may invest time and resources in people other than grandchildren, the present study considers grandchild care because prior studies have shown that it is an extremely important form of intergenerational support (Tanskanen and Danielsbacka, 2019). Moreover, empirical studies on the association between grandparental retirement and grandchild care have been somewhat inconclusive.

Most prior studies that considered the association between grandparents' employment status and grandchild care did not distinguish between retired grandparents and those who do not work for other reasons (such as unemployment or illness) (Aassve et al., 2012; Danielsbacka and Tanskanen, 2012; Di Gessa et al., 2016; Železna, 2018; Wilińska et al., 2019; Zamarro, 2020). In addition, prior studies have almost exclusively used cross-sectional data and compared two different groups of individuals, that is, they have compared the grandchild care of working grandparents with that of non-working grandparents. Little is known about how the *transition to retirement* affects grandchild care. One exception is a pioneering study by Lakomý and Kreidl (2015), who used longitudinal data from 13 European countries and found that the transition from full-time employment to being “out of the labor force” increased the frequency of child care provided by a grandparent over time. Although the study considered person-specific changes in grandparental child care over time, it did not distinguish between retired grandparents and other grandparents who were out of the labor force and thus the study was unable to capture the “unique” effect of retirement on the provision of grandchild care. Feng and Zhang (2018) found a significant increase in provision of grandchild care after the transition to retirement among urban Chinese grandparents. However, it is important to study whether this effect is also present in contemporary Western countries because intergenerational family relations in Europe tends to differ substantially from those in Asia and China (Shwalb and Hossain, 2017; Zhang et al., 2020).

Although grandmothers are predicted to invest more in grandchildren than grandfathers (as discussed above), the magnitude of the retirement effect could be stronger among grandfathers than grandmothers because of their different roles in the labor market; that is to say, men tend to have more permanent positions and full-time jobs than women (OECD, 2018). Hence, after retirement, grandfathers' time resources and opportunities for kin investment are likely to increase more than those of grandmothers. Moreover, as working grandmothers may provide more grandchild care initially, it could be easier for grandfathers to increase their investment in grandchildren as their investment level at baseline tends to be lower. Following this logic, it could also be that after retirement, paternal grandparents will increase their investment in grandchildren more than maternal grandparents. As for paternal grandparents, it could be easier to provide more care if their baseline investment is lower. Alternatively, if grandmothers are more inclined to provide care to their grandchildren than grandfathers and maternal grandparents more inclined than paternal grandparents, one could predict that the transition to retirement will increase the investment of grandmothers and maternal grandparents more than that of grandfathers and paternal grandparents. Whichever way, it is important to consider the potential sex- and lineage-based differences in the associations between retirement and grandchild care.

Here, we study grandparental investment using longitudinal data from 15 European countries. First, we explore whether transition to retirement is associated with increased levels of grandchild care. Second, we analyze the potential sex- and linage-based differences in the associations between transition to retirement and grandparental child care. The methodological contribution of this paper is to study within-person associations, which consider person-specific changes over time and show whether the transition to retirement increases or decreases child care in European grandparents.

## Data and Methods

### Sample

We used longitudinal data drawn from the Survey of Health, Aging and Retirement in Europe (SHARE) of people aged 50 or older who spoke the official language of their country and who were not living abroad or in an institution during the fieldwork period (see Börsch-Supan et al., 2013 for methodological details of SHARE). Computer-assisted personal interviewing was used as the SHARE data collection method. In the present study, the sample included respondents from the first to the sixth wave of SHARE conducted between 2004 and 2015 across 15 European countries (excluding the third wave, which entailed retrospective life history data collection, SHARELIFE). The countries were Austria, Germany, Sweden, the Netherlands, Spain, Italy, France, Denmark, Greece, Switzerland, Belgium, Czech Republic, Poland, Slovenia, and Estonia. Nine countries (Austria, Germany, Sweden, Spain, Italy, France, Denmark, Switzerland, and Belgium) participated in all five waves investigated here, two countries (Czech Republic and the Netherlands) participated in four waves, and four countries (Greece, Poland, Estonia, and Slovenia) participated in three waves.

In performing the analyses, we selected participants with at least one grandchild and those with available data concerning all the variables studied. Participants who were 75 years old or older were excluded from the sample because such individuals rarely work and thus cannot experience the transition to retirement. Moreover, respondents who were already retired, unemployed, chronically ill, homemakers, or in other ways outside paid employment were excluded from the study sample because they could not undergo the changeover from employment to retirement between survey waves. Only respondents who had participated in at least two survey waves were included in the models. The final study sample consisted of 50- to 74-year-old respondents across five SHARE waves and over the 11-year follow-up period between 2004 and 2015.

### Measures

Grandchild care was the dependent variable in this study. SHARE respondents who had at least one grandchild were asked to report whether they had looked after their grandchild(ren) without the parents being present during the time interval since the last interview (in follow-up waves) or during the preceding 12 months (in the wave during which a participant entered SHARE), and if they had, how often (ranging from 1 = *almost daily* to 4 = *less than monthly*). We calculated the mean grandchild care variable by summing and averaging the answers for all adult children who were parents themselves, producing a scale ranging from 0 = *no care* (45% of all person observations), 1 = *less than monthly* (15%), 2 = *almost every month* (14%), 3 = *almost every week* (18%), and 4 = *almost daily* (8%). For instance, if a grandparent had grandchildren via four children and they looked after the first and second child's children almost every month and the third and fourth child's children almost daily, the mean child care was thus 3 = *almost every week*: (2 + 2 + 4 + 4) / 4 = 3. 25% of grandparents provided care neither before nor after they retired. Among those grandparents who looked after their grandchildren at least occasionally, 28% remained in the same category between the study waves.

The employment status of respondents was our main independent variable, and we selected only those older adults whose status was employed or self-employed (0 = *working*) and retired (1 = *retired*). In total, <1% of the respondents reported a transition from “retirement” back to “working,” and they were subsequently excluded from the sample, because our main goal was to investigate the effect of transition from employment to retirement. Additionally, 5% of participants who had retired as a result of illness were excluded from the sample. Based on the transition probabilities, 25% of the participants experienced a transition to retirement between the study waves, with 22% for women and 28% for men.

### Analytic Strategy

To investigate whether the transition to retirement was associated with changes in grandparental childcare, we applied within-person (or fixed effect) regression models where the repeated measures (i.e., person-observations) were nested within respondents. Total (or random effect) regression models include both between-person and within-person variance, meaning that they can rarely provide evidence for causality, because in these models, the unobserved (time constant) heterogeneity is typically not appropriately considered (Ates, 2017). To examine more causal associations between retirement and grandchild care, we excluded between-person variation and concentrated on within-person variation by conducting panel fixed-effect regressions (Curran and Bauer, 2011; Morgan, 2013). Within-person models consider person-specific changes and show an individual's variation over time, i.e., whether transition to retirement increase or decrease the frequency of grandparental child care. As within-person models require variation in the outcome variable (i.e., grandchild care), those respondents who provided identical level of grandchild care between study waves were excluded (Jokela et al., 2018). In the within-person models, the participants served as their own controls, and these models eliminated all time-invariant factors (Allison, 2009; Brüderl and Ludwig, 2015), meaning that factors whose values did not change between the study waves were automatically controlled regardless of whether they were available in the SHARE data (e.g., sex, country of residence, as well as many genetic factors and other selection effects). Our final sample for within-person analyses consisted of 37,394 person observations from 14,964 individuals.

To achieve more robust results, several factors were controlled for in the analyses: respondents' age at interview, partnership status, self-rated health (ranging from 1 = *poor* to 5 = *excellent*), difficulties with basic activities of daily living (ADL limitations, ranging from 0 to 23, where a higher number indicated a higher number of limitations), and number of grandchild sets (i.e., how many of the respondents' children had children). Descriptive statistics of participants included in the analyses of within-person associations are presented in Table 1.

**Table 1:** Descriptive statistics.

	**%**	**Mean (SD)**	**Within-person SD**
Sex			
Female	57.6		
Male	42.4		
Lineage			
Grandchildren via daughters only	24.3		
Grandchildren via daughters and sons	53.6		
Grandchildren via sons only	22.1		
Age at interview		64.4 (5.90)	2.22
Partnership status			
Have a spouse/partner	68.0		
No spouse/partner	32.0		
Self-rated health		3.05 (1.03)	0.51
ADL limitations		1.33 (2.36)	1.17
Number of grandchild sets		2.30 (0.84)	0.19
Country			
Austria	7.3		
Germany	6.8		
Sweden	9.2		
Netherlands	4.1		
Spain	3.9		
Italy	5.3		
France	9.1		
Denmark	8.0		
Greece	2.3		
Switzerland	4.5		
Belgium	9.9		
Czech Republic	12.3		
Poland	2.4		
Slovenia	5.3		
Estonia	9.9		

*n = 37,394 person-observations from 14,964 unique persons*.

We ran several sensitivity analyses related to our within-person models considering the association between transition to retirement and grandchild care. First, although our dependent variable, grandparental child care, was not normally distributed we did not use logit models due to their limitations (Mood, 2010). Instead, we executed sensitivity analyses using logistic regression models with different cut-off points, and for these models we constructed three dichotomous grandparental child care variables: 0 = *no care*, 1 = *at least some care* (including all other classes); 0 = *less often than almost monthly*, 1 = *at least almost monthly*; 0 = *less often than almost every week*, 1 = *almost daily or every week*. Second, to confirm the correct temporal order between the dependent (grandparental child care) and main independent (retirement) variables, we measured grandchild care as forward-lagged (i.e., one wave after retirement). Third, to study reverse causality, we used forward-lagged retirement as the dependent variable and grandchild care as the main independent variable. Fourth, to avoid a drop in the number of observations, the age of the youngest grandchildren and geographical distance between grandparents and adult children were not controlled for in the basic analyses because SHARE only collected this information systematically with regard to the respondents' four oldest children. Instead, sensitivity analyses were performed, which controlled for these variables. Finally, to examine potential cultural differences, the countries were classified into groups (Danielsbacka et al., 2011; Di Gessa et al., 2016), and were used instead of specific countries to avoid any loss of statistical power. The four country groups were Southern Europe (Spain, Italy, and Greece), Central Europe (Austria, Germany, France, Belgium, and Switzerland), Northern Europe (Denmark, the Netherlands, and Sweden), and Eastern Europe (the Czech Republic, Poland, Slovenia, and Estonia). Findings from the sensitivity analyses are presented at the end of the Results section.

In the tables, the magnitudes of the coefficients are presented as β-coefficients from linear or odds ratios from logistic regression models. In the figures, we show the illustrated results by calculating the adjusted means (or predictive margins) and 95% confidence intervals from regression models (see Williams, 2012 for margins command in Stata).

## Results

**Figure 1:**
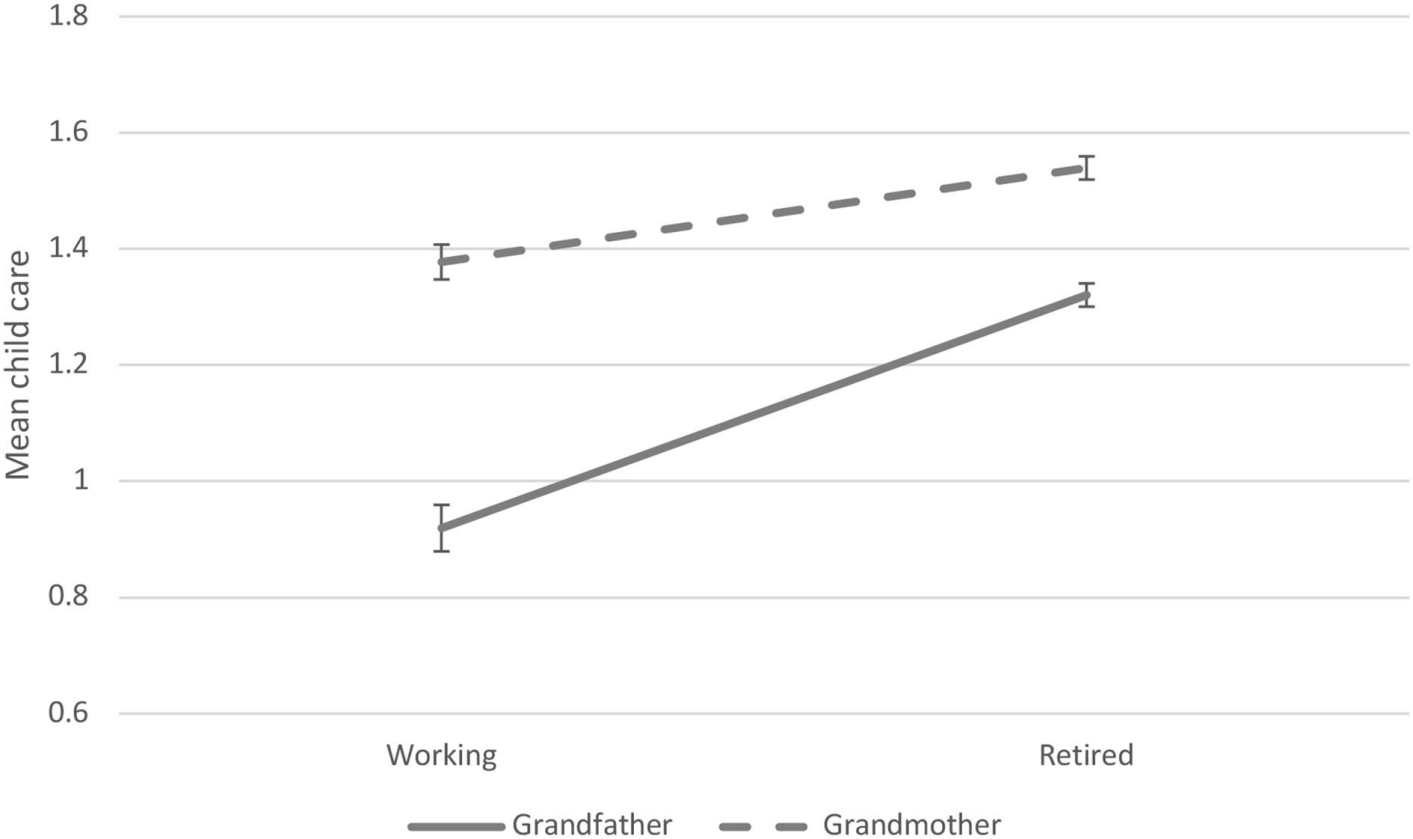
Within-person associations between grandchild care and retirement by sex of grandparent (predictive margins and 95% CIs).

We investigated whether the transition to retirement was associated with the frequency of grandparental child care. Stability in the frequency of grandchild care was relatively high, as indicated by intraclass correlation of 0.64. Overall, we found that the transition to retirement was associated with increased child care (Table 2). When we stratified our data by sex, we found a similar effect in both women and men. However, the interaction model showed that the effect of retirement on child care was stronger among grandfathers than grandmothers (β = 0.17, *SE* = 0.05, *p* < 0.001) (Figure 1). According to lineage, with stratified data, we found that in all groups (i.e., “grandchildren via daughters only,” “grandchildren via daughters and sons,” “grandchildren via sons only”) transition to retirement was associated with increased grandchild care. When those having “grandchildren via daughters only” and those having “grandchildren via sons only” were compared, no significant interaction effect was detected regarding levels of child care (β = 0.08, *SE* = 0.08, *p* = 0.310).

**Table 2:** Within-person associations between retirement and grandparental childcare (stratified by sex and lineage).

				**95% CI**	
	**β**	**SE**	**p**	**lower**	**upper**
**Model 1: All**					
Working	ref				
Retired	0.26	0.03	< 0.001	0.21	0.32
**Model 2: Women**					
Working	ref				
Retired	0.25	0.04	< 0.001	0.18	0.32
**Model 3: Men**					
Working	ref				
Retired	0.28	0.03	< 0.001	0.22	0.34
**Model 4: Grandchildren via daughters only**					
Working	ref				
Retired	0.31	0.06	< 0.001	0.19	0.43
**Model 5: Grandchildren via daughters and sons**					
Working	ref				
Retired	0.26	0.04	< 0.001	0.18	0.33
**Model 6: Grandchildren via sons only**					
Working	ref				
Retired	0.26	0.06	< 0.001	0.14	0.38

*Control variables include respondents' age, partnership status, self-rated health, ADL limitations and number of grandchild sets*.

*Model 1: n = 37,394 person-observations from 14,964 unique persons*.

*Model 2: n = 21,764 person-observations from 8,618 unique persons*.

*Model 3: n = 15,630 person-observations from 6,346 unique persons*.

*Model 4: n = 9,097 person-observations from 3,640 unique persons*.

*Model 5: n = 20,032 person-observations from 8,016 unique persons*.

*Model 6: n = 8,265 person-observations from 3,308 unique persons*.

Given that the grandparental child care variable was not normally distributed, we ran analyses with categorized variables using logistic regression models with three different cut-off points (0 = *no care*, 1 = *at least some care*; 0 = *less often than almost monthly*, 1 = *at least almost monthly*; 0 = *less often than almost every week*, 1 = *almost daily or every week*). The associations between the transition to retirement and increased grandchild care were found in all models (Table 3). These additional analyses were also in line with the main analyses, indicating that the findings can be deemed robust.

**Table 3:** Within-person associations between retirement and grandparental childcare with dichotomous childcare variables.

				**95% CI**	
	**OR**	**SE**	**p**	**lower**	**upper**
**Model 1**					
**Grandparental childcare**					
No care	ref				
At least some care	1.76	0.14	< 0.001	1.50	2.05
**Model 2**					
**Grandparental childcare**					
Less often than almost every month	ref				
At least almost monthly	1.71	0.13	< 0.001	1.47	1.99
**Model 3**					
**Grandparental childcare**					
Less often than almost every week	ref				
Almost daily or every week	1.96	0.17	< 0.001	1.66	2.31

*Control variables include respondents' age, partnership status, self-rated health, ADL limitations and number of grandchild sets*.

*Model 1: n = 12,763 person-observations from 4,728 persons*.

*Model 2: n = 13,161 person-observations from 4,821 persons*.

*Model 3: n = 11,303 person-observations from 4,159 persons*.

Then, we executed sensitivity analyses where we controlled for the mean age of the youngest grandchild and mean geographical distance variables. This analysis provided similar results to those found in the main analyses (β = 0.21, *SE* = 0.04, *p* < 0.001). To confirm the correct temporal ordering between retiring and changes in grandchild care, we ran sensitivity tests where grandparental child care, our outcome variable, was measured one wave after the baseline, that is, when the main independent variable (retirement) and covariates were measured. In this case, in addition to the controls mentioned above, we controlled for the time span (in months) between the study waves (*M* = 30.0, within-person *SD* = 7.89, ranging from 11 to 64 months). The sensitivity analyses with a forward-lagged child care variable provided results similar to those found in the main analyses (β = 0.20, *SE* = 0.04, *p* < 0.001).

We then tested the reverse causality concerning the direction of the association. The question here was: Does the change in grandparental child care increase grandparents' likelihood of retiring? Theoretically, it could be that older adults who are more inclined to look after their grandchildren are willing to retire earlier. The reversed causality hypothesis was investigated using retirement as the dependent variable and grandparental child care as the main independent variable. In this case, to establish the correct temporal ordering, grandparental child care (and covariates) were measured in one study wave before the outcome variable of retirement. It was found that grandparental child care was not a significant predictor of grandparents' entry into retirement (*OR* = 1.22, *SE* = 0.20, *p* = 0.212); thus, support for reversed causality was not evident.

**Figure 2:**
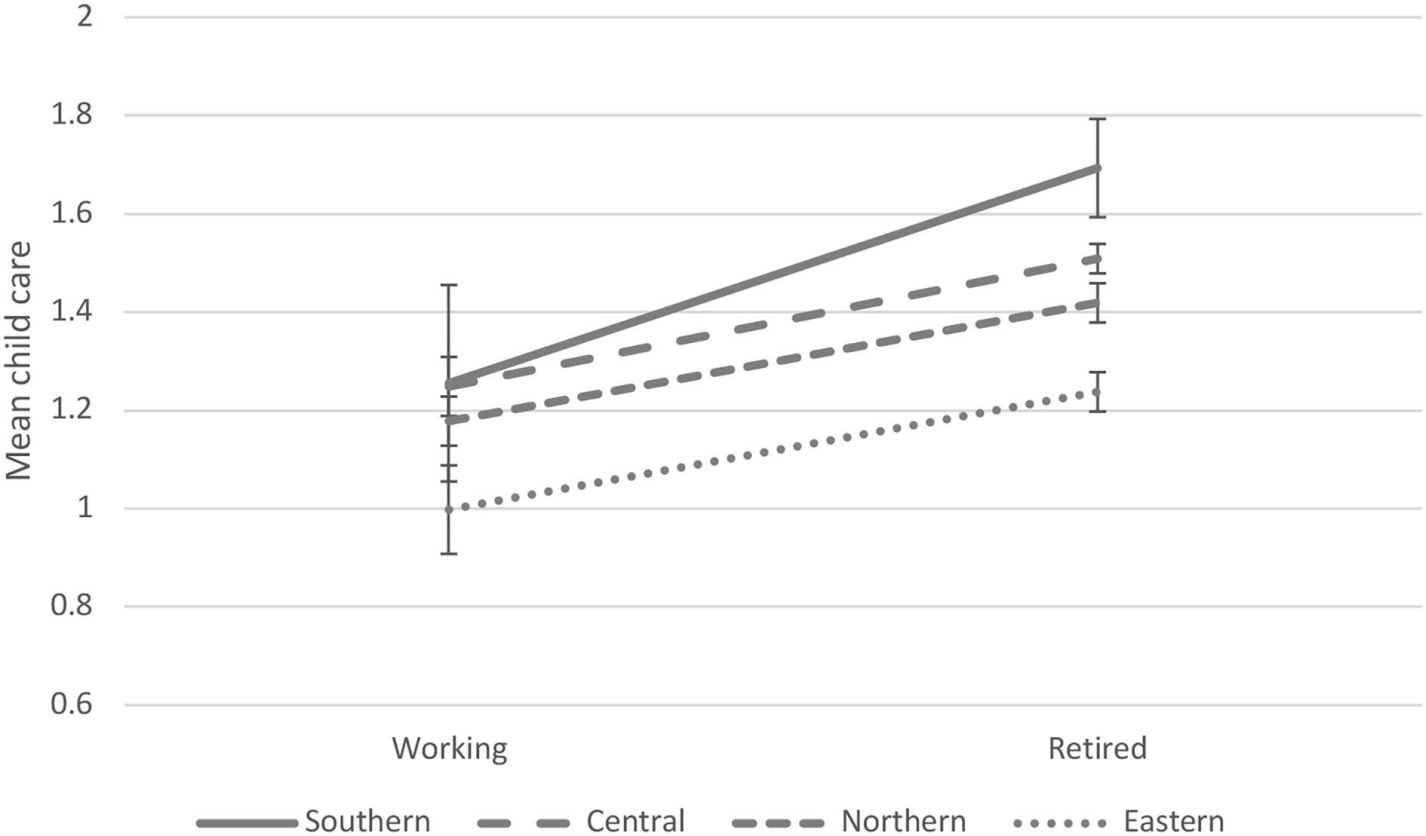
Within-person associations between retirement and grandchild care by European country groups (predictive margins and 95% CI).

Finally, we investigated the potential country differences in the associations between retirement and grandchild care when the countries were grouped into four categories: Southern Europe, Central Europe, Northern Europe, and Eastern Europe. When we stratified the data by country groups, we found that the transition to retirement was associated with increased grandchild care in all country groups (Figure 2). The effect of magnitude was strongest in Southern Europe, followed by Northern Europe, Central Europe, and Eastern Europe. When we included the interaction term between country group and retirement, a significant interaction effect was found (β = −0.11, *SE* = 0.03, *p* < 0.001).

## Discussion

In this article, we examined whether the transition from employment to retirement was associated with changes in the frequency of child care provided by grandparents to their adult children's families. Using within-person regression models we found that grandparents' transition to retirement was associated with increased grandchild care. In addition, we found that the effect of transition to retirement on increased grandchild care was stronger among grandfathers than grandmothers. This finding could be based on the fact that, on average, older men tend to work more hours than older women (OECD, 2018). This also means that transition to retirement may increase older men's time resources (and thus the possibility of investing more time in grandchildren) more than that of older women. Moreover, because grandchild care provided by grandfathers is at a lower level to begin with, it may be easier for older men to increase the frequency of care provided when compared with older women who already invest more intensively in their grand-offspring. It was also found that the transition to retirement was associated with increased grandchild care among both maternal and paternal grandparents. However, there was no significant difference between maternal and paternal grandparents in the magnitude of the effect of transition to retirement on grandchild care. Finally, we detected that the transition to retirement was associated with increased grandchild care in all parts of Europe. Although the magnitude of effect varied between country groups being strongest in Southern Europe and weakest in Eastern Europe, the positive association was evident in all regimes.

To the best of our knowledge, the present study is the first to explore whether transition to retirement affects the frequency of grandchild care within an individual over time using data from Western countries. In line with our findings, a prior study found an increase in provided grandchild care after entry into retirement among urban Chinese grandparents (Feng and Zhang, 2018). Similar to our investigation, Lakomý and Kreidl (2015) found that the transition from full-time employment to part time employment or being out of the labor force in Europe was associated with increased grandchild care over time. As the within-person investigation of Lakomý and Kreidl (2015) focused on transitions between different levels of labor market involvement and did not distinguish retirees from others who are out of the labor force, the study did not estimate the unique effect of retirement on the provision of grandchild care, nor did the study consider possible differences between grandmothers and grandfathers, maternal and paternal grandparents, or between country groups.

The strengths of the present study include the use of large-scale, population-based, cross-national, and longitudinal data, in which the same individuals were interviewed repeatedly. To fully exploit the potential of the panel data, we executed within-person regressions, which consider an individual's variation over time and eliminate all time-invariant factors, making it possible to determine more causal inferences in the association between retirement and grandchild care. Moreover, we were able to control for several time-variant factors available in the SHARE data.

Although within-person models have several strengths, they are not without limitations. First, panel attrition may have influenced the results. Selective panel attrition can exist, for instance, if grandparents who provide most childcare are also most likely to participate in follow-up waves or if grandparents who retire are less likely to participate in follow-up waves. In addition, within-person models may be concerned with the small number of participants who experience changes regarding outcome and main independent variables, meaning that the sample size may decrease and given the low number of observations, within-person models may suffer from high confidence intervals. Although in the main analyses including all countries, we had sufficient observations in the sample, the stratified country group analyses were more likely to suffer from a lack of statistical power. Finally, a limitation of within-person models is that they do not account for time-variant unobserved characteristics. Although we controlled for a wide range of time-variant factors in the models, practically no model can take all of them into account.

The present findings have several practical implications. Prior studies have indicated that grandparental support may positively influence the fertility decisions of adult children (Kaptijn et al., 2010; Tanskanen et al., 2014) and the well-being of grandchildren (Sear and Coall, 2011; but see Tanskanen and Danielsbacka, 2018). Based on our results, retirement can significantly help grandparents become involved in the lives of their descendants, which, in turn, may help younger adults to fulfill their child-bearing plans and improve their well-being. As grandparental child care has been found to increase the labor force participation of mothers with small children (Aassve et al., 2012; Arpino et al., 2014; Bratti et al., 2018; Kanji, 2018; Du et al., 2019), the retirement of grandparents can help parents to combine paid employment and family life. Taking care of grandchildren may also have desirable consequences for the grandparents themselves (Arpino and Bordone, 2014; Danielsbacka and Tanskanen, 2016; Danielsbacka et al., 2019), meaning that retirement may promote the well-being of older adults because they may be able to spend more time with their grandchildren.

Retirees are often considered a social and economic burden to society, for instance, in recent discussions about raising the average retirement age in Europe (European Commission, 2010). Although retirement is regularly perceived as a passive and unproductive phase of life, an increasing number of studies have shown that retired citizens can be socially active, and retirement may even promote social support provided to others (Van den Bogaard et al., 2014; Fischer and Müller, 2020; Grünwald et al., 2021). The present study indicated that when grandparents retire, they tend to provide more grandchild care, which, in turn, may have consequences for their own well-being as well as the well-being of their descendants, as discussed above. Thus, raising the age of retirement may affect not only the older generation but can also have unanticipated repercussions for the younger generations. The fact that retired people can be active and productive citizens and that they are prepared to invest a large amount of time and other resources in their descendants should also be acknowledged more carefully in policymaking and discussions considering the societal role of older people.

## Data Availability Statement

Publicly available datasets were analyzed in this study. This data can be found here: http://www.share-project.org/data-access.html.

## Ethics Statement

The SHARE study is subject to continuous Ethics review. During Waves 1 to 4, SHARE was reviewed and approved by the Ethics Committee of the University of Mannheim. Wave 4 of SHARE and the continuation of the project were reviewed and approved by the Ethics Council of the Max Planck Society. The patients/participants provided their written informed consent to participate in this study.

## Author Contributions

AT, MD, and AS-A designed the study. AT analyzed the data and drafted the manuscript. All authors made a substantial contribution and were involved in writing the paper and interpretation of the results.

## Funding

The present study was supported by the Academy of Finland (grant number 317808 and 320162 and 325857 and 331400).

## Conflict of Interest

The authors declare that the research was conducted in the absence of any commercial or financial relationships that could be construed as a potential conflict of interest.

## Publisher's Note

All claims expressed in this article are solely those of the authors and do not necessarily represent those of their affiliated organizations, or those of the publisher, the editors and the reviewers. Any product that may be evaluated in this article, or claim that may be made by its manufacturer, is not guaranteed or endorsed by the publisher.
